# A knowledge, attitude and practice study of evidence-based nursing combined with narrative nursing mode to improve the quality of life of glioma patients

**DOI:** 10.3389/fmed.2025.1641749

**Published:** 2025-11-19

**Authors:** Jiehui Weng, Lihong Ma, Jianrong Li, Li Zhu, Yanwei Fang

**Affiliations:** 1 Department of Neurosurgery, The Second Hospital of Hebei Medical University, Shijiazhuang, Hebei, China; 2 Department of Emergency Surgery, The Second Hospital of Hebei Medical University, Shijiazhuang, Hebei, China

**Keywords:** glioma, evidence-based nursing, narrative nursing, knowledge-attitude-practice, quality of life

## Abstract

**Background:**

Existing nursing knowledge-attitude-practice (KAP) studies have mostly focused on a single model of evidence-based nursing (EBN) or narrative nursing (NN) rather than an integrative approach. Meanwhile factors influencing nurses’ KAP in glioma care scenarios have not been studied. These gaps hinder the translation of evidence-based and narrative nursing integration into clinical practice and limit the improvement of patient prognosis.

**Methods:**

The knowledge-attitude-practice online questionnaire for nurses included the EBN knowledge questionnaire and NN cognitive assessment. Structural equation modeling was used to analyze the path relationships between knowledge, attitude, and practice. Pearson correlation coefficient was employed to explore the correlations among knowledge, attitude, and practice. Multivariate logistic regression analysis was conducted to examine the impact of demographic factors on the knowledge-attitude-practice levels of nurses. The quality of life of glioma patients was assessed using the European Organization for Research and Treatment of Cancer Quality of Life Questionnaire.

**Results:**

A total of 164 nurses and 352 glioma patients were included in the study. Significant differences in the three knowledge-attitude-practice dimensions of nurses were found based on age, length of work, education background and job title. Professional knowledge played a central role in the construction of attitudes and the transformation of behaviors in nurses. A strong positive correlation was observed between knowledge and practice (*r* = 0.837), while a moderate positive correlation existed between attitude and both knowledge (*r* = 0.481) and practice (*r* = 0.499). Nurses with higher job title (head nurse) performed better in terms of attitude. The impact of education level on the three knowledge-attitude-practice dimensions was relatively small. Additionally, the overall functional status of glioma patients was good (median = 86 points, Q1 = 70.93, Q3 = 95), there was a significant difference in health status (median = 66.7 points, Q1 = 50, Q3 = 83.3), while the difference in symptom severity was relatively small (median = 11.11 points, Q1 = 3.7, Q3 = 24.69).

**Conclusion:**

The knowledge-attitude-practice levels of nurses on EBN and NN are influenced by their own background factors, with professional knowledge playing a crucial role in shaping their attitude and behavior transformation, which may help in developing targeted educational interventions and effective public health strategies for glioma patients.

## 1 Introduction

Glioma is a type of tumor originating from glial cells in the brain, accounting for approximately 80% of all malignant brain tumors (1). The most common and aggressive type of glioma is glioblastoma, which makes up 50% of all malignant brain tumors (2). Glioma is characterized by high invasiveness and recurrence, severely affecting both the survival time and quality of life of patients, with a 5-year survival rate of less than 10% (3, 4). After surgery, glioma patients commonly face a range of physical and psychological issues, including motor dysfunction, speech impairment, cognitive decline, and prevalent feelings of depression and anxiety (5–7). Traditional nursing, however, prioritizes physical rehabilitation over psychosocial support-creating a critical gap in addressing glioma patients’ holistic needs.

Glioma patients’ unique dual burden of physical and psychological morbidity demands comprehensive nursing approaches that integrate evidence-based rigor and patient-centered care. Evidence-based nursing (EBN) leverages the best scientific evidence, clinical expertise, and patient preferences to reduce complications and improve recovery (8, 9), while narrative nursing (NN) centers on listening to patients’ stories to alleviate psychological distress and reconstruct life meaning (10). The combined application of EBN and NN in the care of glioma patients not only addresses post-operative physiological issues through scientific methods but also improves the overall quality of life through psychological counseling, making it an important complement to traditional nursing models (11). However, the promotion of this combined nursing model in clinical practice faces multiple barriers, including insufficient nurse knowledge, low attitude acceptance, and a lack of practical skills.

As the core executors of clinical care, the knowledge, attitude, and practical skills of nurses directly influence the effectiveness of nursing interventions (12, 13). Effective integration of evidence-based and narrative care for glioma care relies on nurses having the knowledge to combine the two, a positive attitude toward holistic care, and the ability to apply these skills in a hyperbaric neurosurgical environment. However, three key research gaps remain: (1) no study has yet assessed the knowledge-attitude-practice (KAP) status of nurses integrating EBN and NN in glioma care; (2) existing nursing KAP research focuses on a single model rather than an integrative approach (14); and (3) factors influencing nurses’ KAP in glioma care scenarios have not yet been examined (15). These gaps impede the translation of EBN and NN integration into clinical practice and limit improvements in patient prognosis. Therefore, this study is based on the following hypotheses: there are significant differences in KAP dimensions among nurses in terms of age, years of experience, title and educational background, nurses’ professional knowledge plays a central role in shaping their attitudes and transforming their behaviors, and good KAP is conducive to the care and rehabilitation of glioma patients.

However, many nurses currently lack a deep understanding of the core concepts of evidence-based nursing and narrative nursing, and even question their actual clinical utility (15). At the same time, challenges (high workloads, insufficient systematic training, and limited practical guidance) make it difficult to promote new nursing models. Therefore, it is crucial to clearly understand the current KAP status of nurses regarding this combined nursing model, analyze its shortcomings and advantages, and provide a scientific basis for subsequent training and management. This is a key step in improving the quality of glioma care. This study aims to quantify and assess the KAP levels of nurses, reveal key factors that influence their nursing practices, and provide solid theoretical and practical support for optimizing nursing education, promoting nursing models, and improving glioma patient outcomes.

## 2 Materials and methods

### 2.1 Study design and participants

This was a cross-sectional study with two relevant cohorts: nurses and glioma patients. The patient cohort was included to initially explore whether nurses’ KAP about evidence-based care-narrative care had potential associations with patients’ quality of life.

Online questionnaires were prepared for nurses. Inclusion criteria: (1) registered nurses from the neurosurgery, oncology, and rehabilitation departments; (2) at least 1 year of clinical nursing experience; (3) documented experience caring for ≥ 5 glioma patients in the past 6 months (verified via nursing shift records). Exclusion criteria: (1) nurses who were not directly involved in clinical care; (2) individuals who were on leave or unable to participate due to health reasons. Inclusion criteria for patients: (1) confirmed diagnosis of glioma via pathological examination; (2) post-operative recovery period with stable vital signs; (3) ability to understand and complete questionnaires; (4) no history of severe psychiatric diseases or other malignant tumors; (5) willingness to provide informed consent. Exclusion criteria for patients: (1) severe post-operative complications (e.g., intracranial infection, cerebral edema) requiring intensive care; (2) language barriers or cognitive impairment preventing questionnaire completion; (3) refusal to participate in research by themselves or their families. In the Second Hospital of Hebei Medical University, Questionnaires for nurses were collected from December 2024 to February 2025, and questionnaires for glioma patients were collected from December 2024 to March 2025. The survey was carried out in two target groups: nurses (*N* = 164) and glioma patients (*N* = 352). This work has been carried out in accordance with the Declaration of Helsinki (2000) of the World Medical Association. This study was approved by the Ethics Committee of the Second Hospital of Hebei Medical University (No. 2020-R588). All participants participated voluntarily and provided informed consent.

### 2.2 Sample size

It was assumed the research population of nurses was 200, with an expected confidence level of 95% and a margin of error of 5%. The sample size calculation yielded approximately 132 individuals. Accounting for a 10% inefficiency, the actual required sample size was at least 150 individuals. Questionnaires were distributed to 164 nurses who met the criteria, with a 100 per cent return rate. This patient cohort analysis was defined as an exploratory study and included glioma patients who met the inclusion and exclusion criteria, aiming to preliminarily explore the potential association between nurse KAP and patient outcomes, without formal hypothesis testing of patient-related priorities. The target candidate patients were 374, 5, 16, and 3 were excluded due to intensive care, postoperative cognitive impairment, and refusal to participate in the study, respectively, resulting in the inclusion of 352 glioma patients.

### 2.3 Survey questionnaire

Based on validated tools, the KAP questionnaire for nurses included the Evidence-Based Nursing Knowledge Questionnaire and the Narrative Nursing Cognition Assessment. The questionnaire design was based on previously reported relevant literature (16, 17). After the initial design, feedback was obtained from 8 oncology nursing experts. Based on their suggestions, the questionnaire was revised, and an internal consistency test for the questionnaire items was conducted. The result showed a reliability coefficient (Cronbach α) of 0.97.

The final questionnaire was an online version in Chinese, consisting of four sections: demographic information (age, length of work, education background, job title, and department), knowledge dimension, attitude dimension, and practice dimension (Supplementary File 1). Each participant took approximately 5 min to complete the online questionnaire. During the process, the research team provided guidance and instructions to ensure that nurses gave informed consent and were made aware of the purpose of study and the confidentiality of the data. The patient filled out the questionnaire when discharged from the hospital. The quality of life of glioma patients was assessed using the European Organization for Research and Treatment of Cancer Quality of Life Questionnaire (EORTC QLQ-C30), which included three main sections: functional scale, symptom scale, and overall health status (Supplementary File 2). The higher scores in the functional domain and overall health status domain represented the better the quality of life. Conversely, higher scores in the symptom domain indicated a poorer quality of life (18).

The EORTC QLQ-C30 consists of 30 items (labeled Q1–Q30), each corresponding to specific functional, symptom, or global health domains (see Supplementary Table 1 for detailed item-domain mapping). Functional scales and symptom scales: All items under these two categories use a 4-point Likert response scale, where responses are coded as follows: 1 = “not at all,” 2 = “a little,” 3 = “somewhat,” and 4 = “a lot.” Global health status scale: This scale includes only two items (Q29 and Q30), which use a 7-point Likert response scale. Responses range from 1 (representing “very poor”) to 7 (representing “very good”). The scores for each dimension were also standardized.

### 2.4 Statistical analysis

The independent variables of the study were the demographic characteristics from participants, including age, length of work, education background, job title, and department. The dependent variable was the KAP scores. Categorical variables were analyzed using *t*-test and ANOVA. The qualification rate for the KAP scores was calculated based on 60% of the total score (19). Chi-square tests were employed for difference analysis to compare the score differences between different variables. Structural equation modeling was applied to analyze the path relationships between the KAP behaviors of healthcare workers in the context of EBN combined with NN. The KAP Structural Equation Model (SEM) is an empirical analysis model constructed based on the KAP theoretical framework (Knowledge, Attitude, Practice). By integrating the “measurement model” and the “structural model,” the causal relationship among knowledge, attitude and behavior is quantitatively analyzed, and at the same time, the measurement validity of the scale items on latent variables is verified.

The convergent validity was assessed using factor loadings (20). In this study, exploratory factor analysis was conducted using the diagonal weighted least squares (DWLS) method to estimate factor loadings by weighted minimization residuals. Factor loading ≥ 0.4: basic convergent validity; ≥ 0.5: good convergent validity; ≥ 0.7: excellent convergent validity. Pearson correlation coefficients were used to explore the relationships between knowledge, attitude, and practice. Multivariate linear regression analysis was conducted to assess the impact of demographic factors on KAP levels. All statistical analyses were conducted using R language (version 4.4.3), and a two-sided *P* < 0.05 was considered as statistically significant.

## 3 Results

### 3.1 Participant characteristics

The basic characteristics of the nursing population were displayed in Table 1, including age, length of work, education background, job title, and department. The results revealed that the majority of the nurses in this survey were aged between 30 and 40 years (69.51%), indicating that the participants were predominantly middle-aged and young nurses. Nurses under 30 years and over 40 years old accounted for 15.85% and 14.63%, respectively. In terms of length of work, the largest group of nurses had 11–15 years of experience (48.78%), followed by 6–10 years (21.34%), ≥ 16 years (17.68%), and 1–5 years (12.20%). Regarding education background, the highest proportion was undergraduate course (89.02%). Concerning job title, the vast majority were frontline clinical nurses (96.34%), while only 3.66% hold the position of head nurse in a ward. As for the department, nearly all of the nurses worked in neurosurgery (99.39%), with a small minority working in rehabilitation (0.61%). Supplementary Table 2 demonstrates the baseline characteristics of the patients, including age, sex, and tumor stage, with the majority of patients with gliomas having a WHO grade of 4 (66.2%).

**TABLE 1:** Baseline characteristics of nurses (*N* = 164).

Characteristic		N (%)
**Age (years)**		
	< 30	26 (15.85)
	30–40	114 (69.51)
	> 40	24 (14.63)
**Length of work (years)**		
	1–5	20 (12.20)
	6–10	35 (21.34)
	11–15	80 (48.78)
	≥ 16	29 (17.68)
**Education background**		
	Junior college	11 (6.71)
	Undergraduate course	146 (89.02)
	Postgraduate and above	7 (4.27)
**Job title**		
	Nurse	158 (96.34)
	Head nurse	6 (3.66)
**Department**		
	Neurosurgery department	163 (99.39)
	Rehabilitation department	1 (0.61)

### 3.2 Analysis of the three dimensions of KAP

The results of the descriptive statistical analysis were presented in Table 2. Except one nurse in rehabilitation department, differential analysis was carried out among three dimensions of KAP in 163 nurses (Table 3). The results indicated that nurses aged below 30 and above 40 years had a higher qualification rate in the knowledge dimension (100% vs. 95.61%) and practice dimension (100% vs. 97.37%) compared to nurses aged 30–40 years (*P* < 0.001). However, nurses aged 30–40 years exhibited the highest qualification rate in the attitude dimension (92.98%, *P* < 0.001). Notably, nurses under 30 years achieved a 100% qualification rate in both the knowledge and practice dimensions, but their qualification rate in the attitude dimension was lower (92.31%), suggesting that although younger nurses possessed strong knowledge and behavioral execution, there was still room for improvement in their alignment with values. Regarding length of work, nurses with 1–5 years and ≥ 16 experience had a 100% qualification rate in the knowledge and practice dimensions (*P* < 0.001). However, nurses with 1–5 years of experience had the lowest qualification rate in the attitude dimension (90%, *P* < 0.001), indicating that more experienced nurses were more likely to understand patient needs and recognized the importance of evidence-based and narrative care, leading to a more positive attitude. In terms of education level, nurses with a diploma or graduate degree or higher achieved a 100% qualification rate in all three KAP dimensions, surpassing those with a junior college degree (*P* < 0.01). Regarding job title, head nurses had a 100% qualification rate in all three dimensions, higher than that of general nurses (*P* < 0.05), suggesting that nurses in management roles demonstrated greater consistency and guidance in both theoretical knowledge and clinical practice. Their experience and sense of responsibility may drive them to place more emphasis on integrating evidence-based and humanistic care in their work.

**TABLE 2:** Descriptive statistical analysis of the three dimensions of KAP for nurses.

Dimension	N	Mean	SD	Median	Minimum	Maximum	Range	Skewness	Kurtosis
Knowledge	163	81.98	12.36	84	45	105	60	−0.07	−0.08
Attitude	163	45.16	6.41	44	26	65	39	0.61	1.24
Practice	163	71.11	9.44	71	44	90	46	−0.08	0.43

KAP, knowledge-attitude-practice; SD, standard deviation.

**TABLE 3:** Analysis of the differences among the three dimensions of KAP in nurses.

Variable		Knowledge				Attitude				Practice			
		Qualified (N)	Total (N)	Qualification rate (%)	*P*	Qualified (N)	Total (N)	Qualification rate (%)	*P*	Qualified (N)	Total (N)	Qualification rate (%)	*P*
**Age (years)**													
	< 30	26	26	100	< 0.001	24	26	92.31	< 0.001	26	26	100	< 0.001
	30–40	109	114	95.61	< 0.001	106	114	92.98	< 0.001	111	114	97.37	< 0.001
	> 40	24	24	100	< 0.001	22	24	91.67	< 0.001	24	24	100	< 0.001
**Length of work (years)**													
	1–5	20	20	100	< 0.001	18	20	90	< 0.001	20	20	100	< 0.001
	6–10	34	35	97.14	< 0.001	32	35	91.43	< 0.001	34	35	97.14	< 0.001
	11–15	76	80	95	< 0.001	75	80	93.75	< 0.001	78	80	97.5	< 0.001
	≥ 16	29	29	100	< 0.001	27	29	93.1	< 0.001	29	29	100	< 0.001
**Education background**													
	Junior college	11	11	100	< 0.001	11	11	100	< 0.001	11	11	100	< 0.001
	Undergraduate course	141	146	96.58	< 0.001	134	146	91.78	< 0.001	143	146	97.95	< 0.001
	Postgraduate and above	7	7	100	0.008	7	7	100	0.008	7	7	100	0.008
**Job title**													
	Nurse	153	158	96.84	< 0.001	146	158	92.41	< 0.001	155	158	98.1	< 0.001
	Head nurse	6	6	100	0.014	6	6	100	0.014	6	6	100	0.014

KAP, knowledge-attitude-practice. The five levels of agreement for the nurse’ responses (strongly disagree, disagree, neutral, agree, strongly agree) were assigned scores ranging from 1 to 5. A score that constitutes 60% of the total score was considered passing. Due to the fact that only one participant from the rehabilitation department took part in the survey, this department was excluded from the analysis.

### 3.3 Structural equation model based on KAP

**FIGURE 1:**
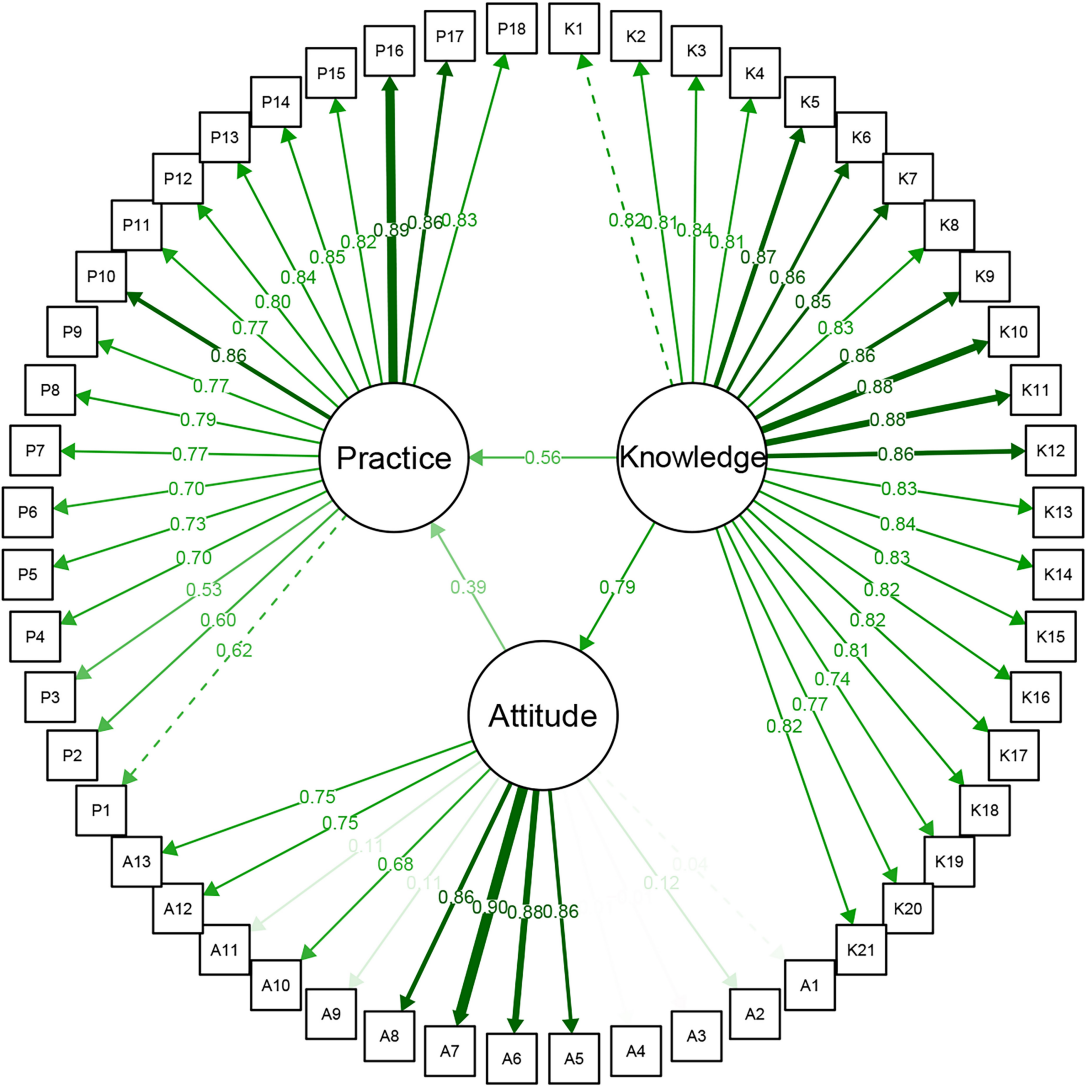
Structural equation model based on KAP for nurses. KAP, knowledge-attitude-practice.

The structural equation model based on KAP was established (Figure 1). The model fit indices Comparative Fit Index (CFI) is 0.941, Tucker-Lewis Index (TLI) is 0.927, Root Mean Square Error of Approximation (RMSEA) is 0.111, Standardized Root Mean Square Residual (SRMR) was 0.028, indicating a good model fit. The results indicated that the path coefficient of knowledge to attitude was 0.79, showing a highly significant positive influence. This suggested that the mastery of professional knowledge (EBN methods and NN skills) was a decisive factor in fostering a positive nursing attitude. Additionally, the direct path coefficient of knowledge to practice was 0.56, further emphasizing that knowledge not only indirectly influences practice through attitude but can also directly drive the behavioral transformation of nursing staff. In contrast, the path coefficient of attitude to practice was 0.39, which, while significant, was relatively weak. This indicated that even if nursing staff had a high positive attitude, their clinical practice transformation may still be limited if they lack adequate knowledge or practical experience. These findings highlighted the central role of knowledge in the KAP model.

In the latent variable measurement model, the majority of the 21 observed variables for the knowledge dimension exhibited factor loadings greater than 0.75, indicating good convergent validity (Supplementary Table 3). The content measured by these items covers key knowledge elements of EBN and NN, reflecting the comprehensiveness and professionalism of healthcare workers in the construction of their professional competence. In the practice dimension, the 18 items also demonstrated a stable factor structure, with overall factor loadings mostly exceeding 0.7. This suggests that the questionnaire effectively measures the practice strategies and coping mechanisms employed by healthcare workers in real-world work scenarios, exhibiting strong construct validity and structural fit. Regarding the attitude dimension, although some items effectively captured the sense of identification and subjective inclination from healthcare workers toward new nursing models, certain negatively worded items showed lower factor loadings. This may be due to the wording of the items or individual interpretation biases, which could reduce the explanatory power of the attitude latent variable.

### 3.4 Correlation among three dimensions of KAP

The research findings revealed a significant positive correlation between knowledge, attitude, and practice regarding the EBN combined with narrative care model (Table 4) (*P* < 0.001). Among these, the correlation between knowledge and practice was the strongest (*r* = 0.837), suggesting that the more thoroughly nurses master the knowledge of this model, the higher their level of clinical practice. Additionally, moderate positive correlations were observed between knowledge and attitude (*r* = 0.481) and between attitude and practice (*r* = 0.499), indicating that attitude may play a mediating role in the translation of knowledge into practice.

**TABLE 4:** KAP correlation for nurses.

Variable pair	Correlation coefficient (r)	95% CI	*P*	Strength of association
Knowledge-attitude	0.481	0.354–0.591	< 0.001	Moderate positive correlation
Knowledge-practice	0.837	0.784–0.877	< 0.001	Very strong positive correlation
Attitude-practice	0.499	0.375–0.606	< 0.001	Moderate positive correlation

KAP, knowledge-attitude-practice; CI, confidence interval.

### 3.5 Multivariate regression analysis in the three dimensions of KAP

Multiple regression analyses of the three dimensions of KAP were conducted to explore the influence of nurses’ demographic and occupational factors on the three dimensions of KAP based on EBN combined with NN (Table 5). In the attitude dimension, no significant difference in knowledge levels was found for work experience (*P* > 0.05). Moreover, the effect of job title on the attitude dimension was not significant (*P* > 0.05). In contrast, a higher level of education (postgraduate and above) had a significant effect on the knowledge dimension (*P* = 0.042), indicating that the education level of nurses has a substantial impact on their attitude acquisition.

**TABLE 5:** Multivariate regression analysis in the three dimensions of KAP for nurses.

Variable	Beta_CI (95% CI)	*P*
**Attitude**		
Length of work (6–10 years)	2.159 (−1.39 to 5.707)	0.231
Length of work (11–15 years)	2.439 (−0.717 to 5.596)	0.129
Length of work (≥ 16 years)	1.209 (−2.567 to 4.984)	0.528
Job title (head nurse)	1.092 (−4.828 to 7.012)	0.716
Education background (postgraduate and above)	6.098 (0.623–11.573)	0.029
Education background (undergraduate course)	2.917 (−1.048 to 6.881)	0.148
**Knowledge**		
Length of work (6–10 years)	−6.136 (−12.915 to 0.643)	0.076
Length of work (11–15 years)	−4.665 (−10.696 to 1.366)	0.129
Length of work (≥ 16 years)	−8.914 (−16.128 to 1.701)	0.016
Job title (head nurse)	−6.707 (−18.017 to 4.603)	0.243
Education background (postgraduate and above)	10.869 (0.409–21.33)	0.042
Education background (undergraduate course)	6.104 (−1.47 to 13.678)	0.113
**Practice**		
Length of work (6–10 years)	−3.955 (−9.163 to 1.253)	0.136
Length of work (11–15 years)	−4.659 (−9.292 to 0.025)	0.049
Length of work (≥ 16 years)	−5.72 (−11.263 to 0.178)	0.043
Job title (head nurse)	−1.341 (−10.03 to 7.349)	0.761
Education background (postgraduate and above)	7.8 (−0.238 to 15.836)	0.057
Education background (undergraduate course)	3.364 (−2.456 to 9.183)	0.255

KAP, knowledge-attitude-practice.

In the knowledge dimension, nurses with ≥ 16 years (*P* = 0.016) of work experience exhibited significantly lower knowledge scores. This indicates that as the number of years of service increases, nurses are less likely to learn new knowledge. The significant effect of education level on knowledge (*P* = 0.042) indicates that the level of education has a significant effect on knowledge acquisition of nurses.

In the practice dimension, no significant difference was observed for nurses with 6–10 years of work experience (*P* > 0.05). However, nurses with 11–15 years (*P* = 0.049) and ≥ 16 years (*P* = 0.043) of work experience showed significantly lower scores in the practice dimension. This suggests that long-term nurses may face challenges in adapting to new nursing methods. Furthermore, neither job title nor education level significantly affected practice scores (*P* > 0.05), indicating that these factors did not significantly influence the performance of nurses in practice. These findings provide valuable guidance for developing more targeted training programs and strategies to improve nursing quality, particularly in addressing knowledge updating and attitude adjustment among experienced nurses.

### 3.6 The quality of life for glioma patients

**FIGURE 2:**
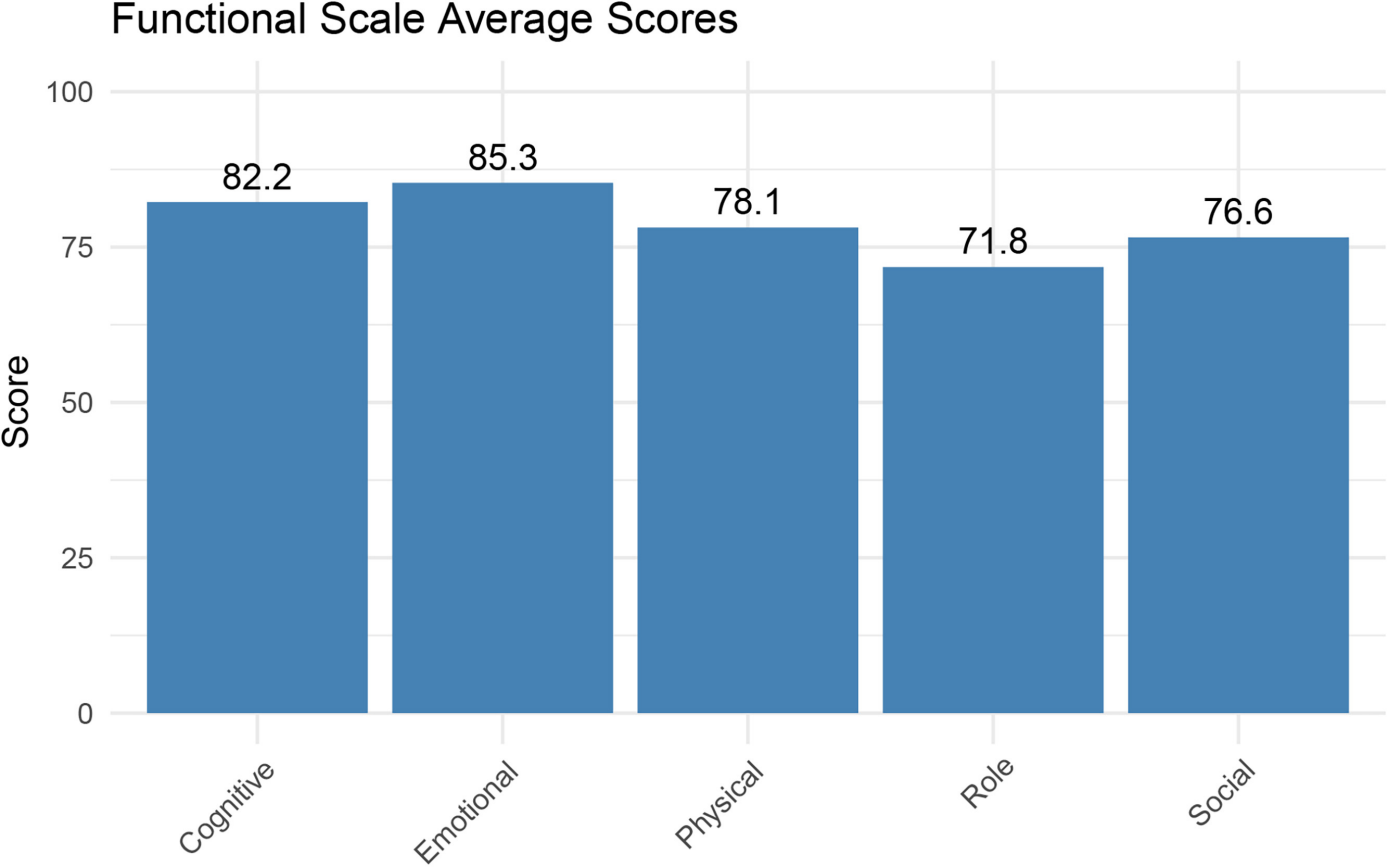
The average score of the functional scale of all patients.

After the combination of EBN and NN on glioma patients, the quality of life for glioma patients was analyzed using the EORTC QLQ-C30 questionnaire, including functional scale, symptom scale, and overall health status. Figure 2 presented the average scores of 5 key functional domains based on the EORTC QLQ-C30 questionnaire. Emotional functioning ranked the highest with an average score of 85.3, indicating that the emotional state of glioma patients was relatively stable. The average scores for cognitive functioning (82.2) and physical functioning (78.1) were also maintained at high levels, suggesting that the cognitive abilities of patients and daily activity levels were relatively normal. Social functioning (76.6) had a moderate average score, showing no severe deficits, but still highlighting potential challenges patients may face in social interactions. Role functioning (71.8) had the lowest average score, reflecting significant barriers for patients in fulfilling their regular social roles, which may be related to their disease status.

**FIGURE 3:**
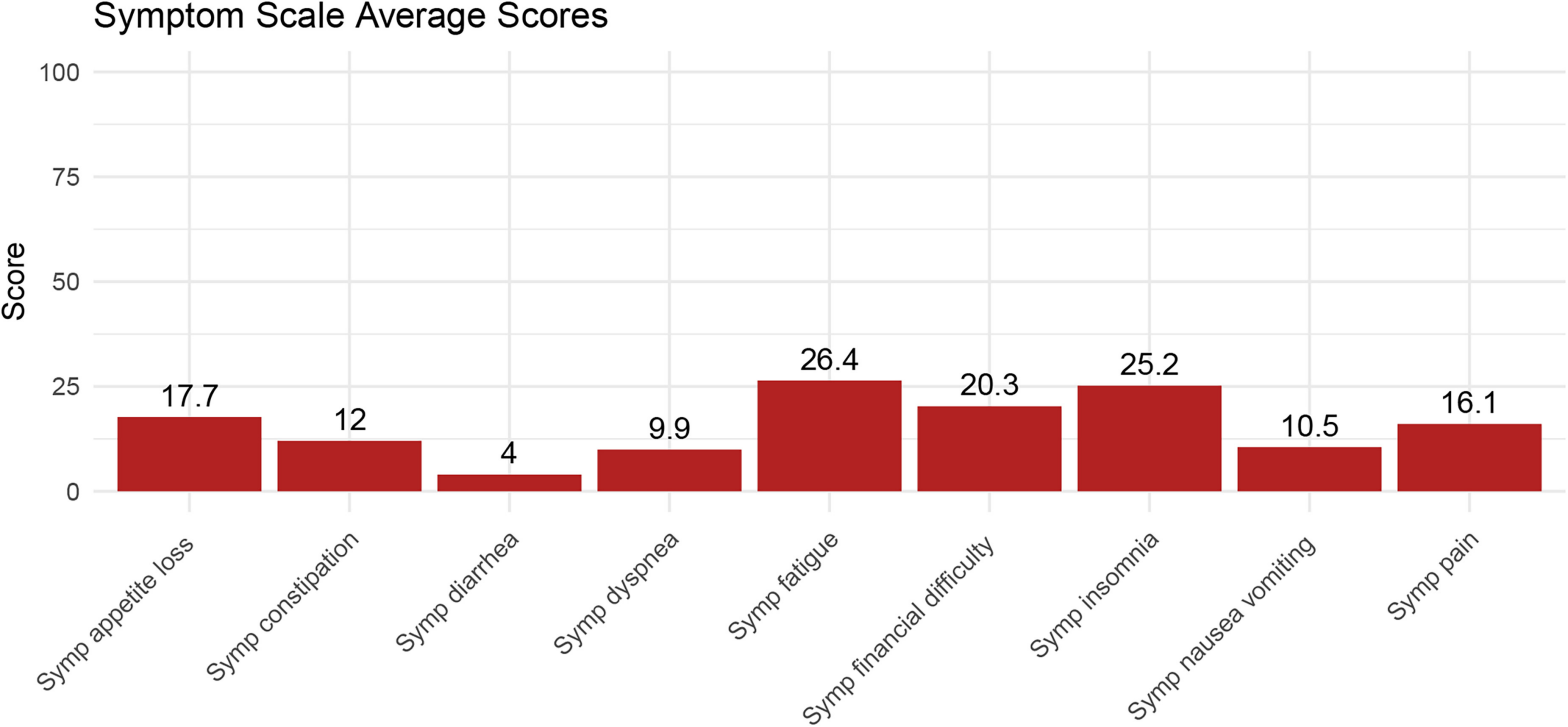
The average score of the symptom scale for all patients.

Figure 3 yielded the overall average scores for various symptoms in glioma patients. The top three symptoms with the highest average scores were fatigue (26.4), insomnia (25.2), and financial difficulty (20.3), indicating that the majority of patients had symptoms of fatigue, insomnia, and financial hardship. However, cases of diarrhea (4) and nausea/vomiting (10.5) are relatively rare.

**FIGURE 4:**
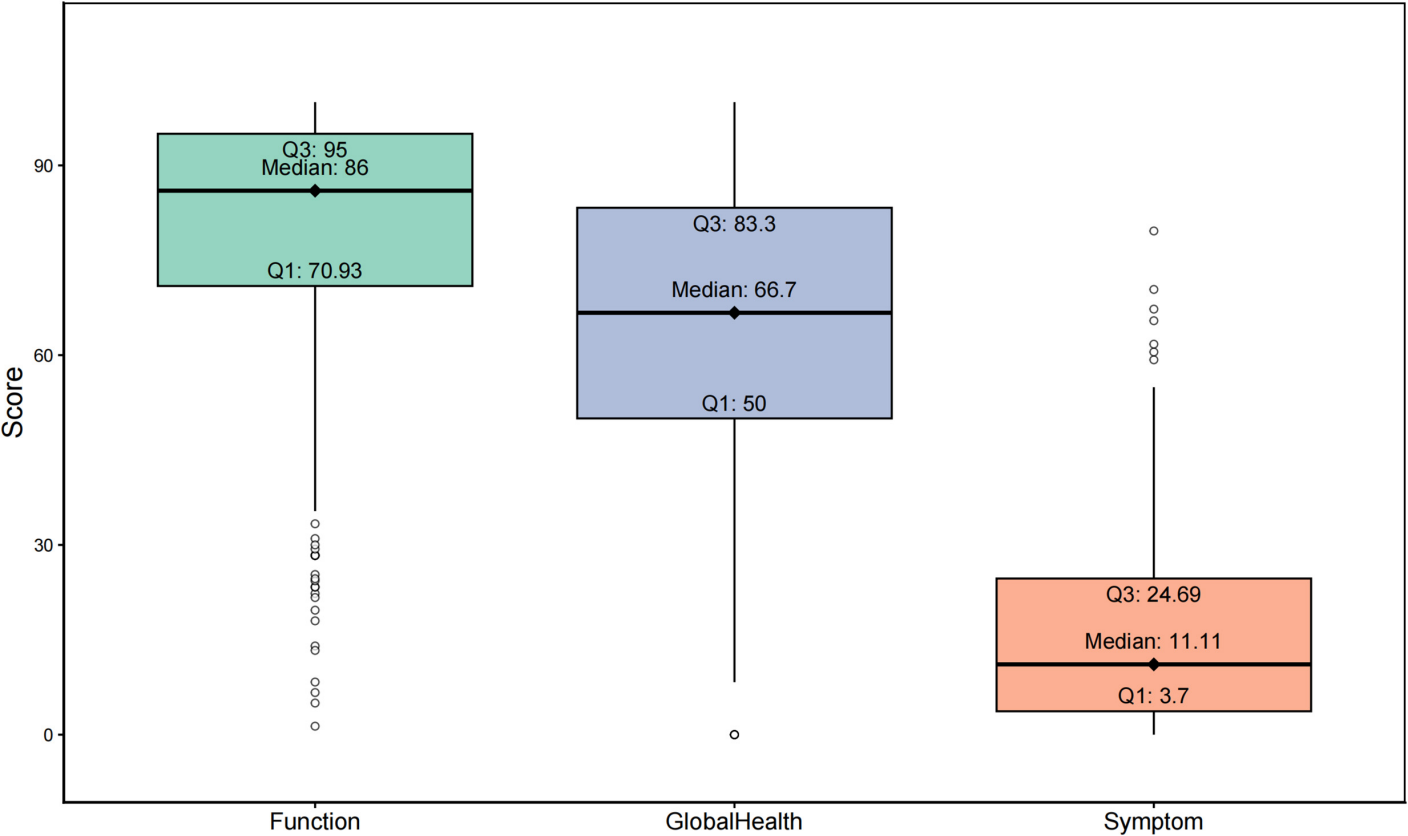
Distribution of function scale, symptom scale and overall health of all patients.

Figure 4 illustrated the distribution of the functional scale, symptom scale, and global health of glioma patients. The median of the functional scale was 86, Q1 and Q3 were 70.93 and 95, respectively, indicating that most glioma patients had good and stable functional status. The median of the overall health scale was 66.7, and Q1 and Q3 were 50 and 83.3, respectively, suggesting that there were some differences in overall health status of glioma patients. The median symptom scale score was 11.11, with a small interquartile range, indicating similar symptom severity in patients with different gliomas. These results indicate that the functional status of glioma patients is generally good and stable, the global health status is moderate and fluctuant, and the severity of symptoms is similar.

## 4 Discussion

Through a cross-sectional analysis of 164 nurses and 352 glioma patients, this study showed that under the combination of EBN and NN, the knowledge-attitude-practice level of nurses is affected by their personal background factors (age, working years, education background and professional title), and professional knowledge plays a key role in attitude and behavior change. These results confirmed that nurses’ KAP in the integration of evidence-based nursing and narrative nursing was modifiable, and KAP was found to be beneficial to the prognosis of patients through preliminary exploration, thus filling the research gap between nurse training and patient care in glioma nursing research.

The demographic background of the nurse population is analyzed in this study. The majority of participants are between 30 and 40 years old, with the highest proportion having more than 6 years of work experience. Most nurses have at least a bachelor degree and are primarily from the neurosurgery department. These basic characteristics provide important background support for further analysis in the KAP levels of nurses under the combined EBN and NN model. KAP surveys are typically conducted through questionnaires, aiming to identify the knowledge, attitude, and practice of the target population, and to uncover any gaps, thereby providing a basis for formulating effective interventions (12, 21). The results of this study show significant differences in the three KAP dimensions of nurses based on their age, years of work experience, job title, and educational background. The differences in KAP levels among these nurse groups may partly be attributed to education level and professional status, as other studies have shown a certain correlation between knowledge and education (22, 23). These findings provide data support for the subsequent development of targeted training plans and management strategies.

The KAP theory demonstrates that knowledge is the foundation of practice, while attitude is the driving force behind practice (24–26). The KAP levels of nurses regarding gliomas vary across different countries (12). The results of this study indicate that the professional knowledge of nurses plays a central role in shaping their attitude and transforming behavior. Furthermore, there is a strong positive correlation between knowledge and practice in nurses, with attitudes indirectly influencing their practices. These results suggest that when nursing staff have a deep understanding of EBN and NN knowledge, they are more likely to develop a proactive clinical attitude, which can be transformed into effective nursing behaviors. Meanwhile, this, promotes the comprehensive physical and psychological rehabilitation of glioma patients, which has significant theoretical and practical value. In addition, years of experience, job title, and educational background may influence the KAP levels of nurses (27–29). This study supports that nurses with longer work experience (≥ 16 years) score lower in knowledge and practice, indicating the need for regular training and motivational measures to help them adapt to new nursing models. Nurses with higher job title (head nurse) perform better in terms of attitudes, likely because they have more opportunities to be exposed to new ideas. Education level has a relatively smaller impact on the three dimensions of KAP, suggesting that educational background has a limited influence on KAP of nurses.

In the daily care of glioma patients, targeted teaching, implementation and outcome-based assessment for nurses are helpful to transform knowledge into practice. Nurses can carry out experiential learning to enhance their ability to use narrative nursing, and NN can be integrated into routine patient care, including bedside narrative, narrative nursing records, etc. These methods can solve the obstacles such as “lack of time” that nurses generally face. At the same time, hospitals can track the frequency of NN conversations carried out by nurses through nursing record review, and measure the change of patients’ emotional distress and the improvement of doctor-patient relationship through satisfaction survey. Together, these constitute a practical framework for the application of NN in the care of glioma patients, which complements the rigor of EBN to provide comprehensive support to patients.

Currently, research on the combined effect of EBN and NN on improving the quality of life of glioma patients is relatively scarce. Existing studies involving other cancer patients indicate that EBN can effectively improve quality of life, reduce symptom burden, and increase satisfaction (30, 31). Additionally, NN can improve the psychological health and quality of life of cancer patients (32). These findings suggest that both EBN and NN play positive roles in the overall care of cancer patients and may have a similar positive impact on the quality of life of glioma patients. The clinically important thresholds (TCIs) for physical functioning, role functioning, social functioning, emotional functioning, and cognitive functioning are 83, 58, 58, 71, and 75 points, respectively (33). In this study, the average scores of cognitive functioning and emotional functioning in patients were higher than the corresponding TCIs, indicating that the patients had no clinically significant cognitive impairment or emotional distress. This finding may be attributed to two key factors: first, the patients were in the postoperative recovery period with relatively stable disease conditions; second, the patients received psychological support from NN, which enhanced their psychological adaptability to the disease. Meanwhile, the average score of physical functioning was below its TCI, suggesting that the patients had mild limitations in daily activities. Although these limitations did not reach the level of severe functional impairment, they have approached the clinical threshold requiring attention—likely a result of incomplete recovery of motor function in the short postoperative period (e.g., surgery-related limb movement restrictions) or treatment-related fatigue affecting physical activity capacity. Regarding social and role functioning, their average scores were higher than the TCIs but ranked the lowest among all functional dimensions. This indicates that while the patients could maintain basic social activities, they still faced difficulties in fulfilling work and family roles. Such challenges may stem from concerns about postoperative conditions, limitations in physical functioning, or uncertainty about disease prognosis, all of which contribute to reduced role adaptation ability. Postoperative interventions for patients should focus on physical functioning, postoperative rehabilitation, and role adaptation support and social integration guidance to strengthen role functioning.

This study has several limitations. Firstly, the subjectivity of self-reported data may lead to deviations in research results. Nurses’ practice scores rely on self-reports and may be overestimated. Similarly, patients’ quality of life scores can be influenced by their emotions on the day of the survey and data omissions. Secondly, the research results are limited to the KAP level of nursing staff in a single hospital and may not represent the level of other nursing teams. Moreover, the improvement in patients’ quality of life is only a speculative conclusion. Thirdly, the cross-sectional design makes it impossible for us to establish a causal relationship. In the future, longitudinal studies can be conducted to track the changes in nurses’ KAP and the corresponding changes in patients’ quality of life, thereby strengthening the causal relationship argument. Finally, there may be bias in sample selection. The inclusion subjects excluded novice nurses and patients with cognitive impairment, which might affect the result analysis. Subsequent studies should include a larger population and adopt the propensity score matching method to balance the sample characteristics.

## 5 Conclusion

In summary, this KAP study about EBN and NN in nurses highlights that age, length of work, job title, and educational background significantly influence the scores of three KAP dimensions. The professional knowledge of nurses plays a central role in shaping their attitudes and transforming their behaviors. These findings are valuable for developing targeted training and intervention measures, ultimately improving the quality of care for glioma patients and enhancing their quality of life.

## Data Availability

The original contributions presented in this study are included in this article/Supplementary material, further inquiries can be directed to the corresponding author.

## Supplementary material

Supplementary File 1 Questionnaire for nurses.

Supplementary File 2 Questionnaire for glioma patients.
