# Supplementary material for: A knowledge, attitude and practice study of evidence-based nursing combined with narrative nursing mode to improve the quality of life of glioma patients
Source: Front Med (Lausanne). 2025 Nov 19;12:1641749. doi: 10.3389/fmed.2025.1641749 (PMC12672325; doi:10.3389/fmed.2025.1641749)
Supplement: Supplementary file 1 [file Table_3.docx]

**Supplementary table 3 Factor loadings of each variable.**

| Variable | Dimension | Factor loading |
| --- | --- | --- |
| Attitude | A1 | 0.038 |
|  | A2 | 0.117 |
|  | A3 | -0.009 |
|  | A4 | 0.009 |
|  | A5 | 0.858 |
|  | A6 | 0.877 |
|  | A7 | 0.901 |
|  | A8 | 0.863 |
|  | A9 | 0.108 |
|  | A10 | 0.675 |
|  | A11 | 0.109 |
|  | A12 | 0.751 |
|  | A13 | 0.755 |
| Knowledge | K1 | 0.82 |
|  | K2 | 0.812 |
|  | K3 | 0.842 |
|  | K4 | 0.814 |
|  | K5 | 0.866 |
|  | K6 | 0.857 |
|  | K7 | 0.854 |
|  | K8 | 0.83 |
|  | K9 | 0.861 |
|  | K10 | 0.88 |
|  | K11 | 0.876 |
|  | K12 | 0.864 |
|  | K13 | 0.833 |
|  | K14 | 0.842 |
|  | K15 | 0.83 |
|  | K16 | 0.822 |
|  | K17 | 0.825 |
|  | K18 | 0.807 |
|  | K19 | 0.745 |
|  | K20 | 0.773 |
|  | K21 | 0.819 |
| Practice | P1 | 0.617 |
|  | P2 | 0.601 |
|  | P3 | 0.535 |
|  | P4 | 0.697 |
|  | P5 | 0.726 |
|  | P6 | 0.704 |
|  | P7 | 0.771 |
|  | P8 | 0.791 |
|  | P9 | 0.765 |
|  | P10 | 0.862 |
|  | P11 | 0.769 |
|  | P12 | 0.8 |
|  | P13 | 0.843 |
|  | P14 | 0.846 |
|  | P15 | 0.825 |
|  | P16 | 0.893 |
|  | P17 | 0.859 |
|  | P18 | 0.832 |
