# Supplementary material for: A knowledge, attitude and practice study of evidence-based nursing combined with narrative nursing mode to improve the quality of life of glioma patients
Source: Front Med (Lausanne). 2025 Nov 19;12:1641749. doi: 10.3389/fmed.2025.1641749 (PMC12672325; doi:10.3389/fmed.2025.1641749)
Supplement: Supplementary File 1 — Questionnaire for nurses. [file Table_1.docx]

**Supplementary table 1: Item Classification of the EORTC QLQ-C30.**

| Scale category | Subscale | Corresponding questionnaire items (Q1–Q30) |
| --- | --- | --- |
| Functional scales | Physical functioning | Q1, Q2, Q3, Q4, Q5 |
|  | Role functioning | Q6, Q7 |
|  | Emotional functioning | Q21, Q22, Q23, Q24 |
|  | Cognitive functioning | Q20, Q25 |
|  | Social functioning | Q26, Q27 |
| Symptom scales | Fatigue | Q10, Q12, Q18 |
|  | Nausea and vomiting | Q14, Q15 |
|  | Pain | Q9, Q19 |
|  | Dyspnea | Q8 |
|  | Insomnia | Q11 |
|  | Appetite loss | Q13 |
|  | Constipation | Q16 |
|  | Diarrhea | Q17 |
|  | Financial difficulties | Q28 |
| Global health status | Global health status | Q29, Q30 |

All items (Q1–Q30) of the EORTC QLQ-C30 are included in the classification. Scoring follows EORTC guidelines: higher scores in functional scales and global health status indicate better quality of life, while higher scores in symptom scales indicate more severe symptom burden.
