# Supplementary material for: A knowledge, attitude and practice study of evidence-based nursing combined with narrative nursing mode to improve the quality of life of glioma patients
Source: Front Med (Lausanne). 2025 Nov 19;12:1641749. doi: 10.3389/fmed.2025.1641749 (PMC12672325; doi:10.3389/fmed.2025.1641749)
Supplement: Supplementary File 2 — Questionnaire for glioma patients. [file Table_2.docx]

**Supplementary table 2 Baseline characteristics of patients (N=352).**

| Characteristic | level | Overall |
| --- | --- | --- |
| n |  | 352 |
| Age (mean (SD)) |  | 48.69 (14.50) |
| Gender (%) | Female | 124 (35.2) |
|  | Male | 228 (64.8) |
| Tumor grading (%) | NOS | 2 (0.6) |
|  | WHO 2 | 21 (6.0) |
|  | WHO 2-3 | 23 (6.5) |
|  | WHO 3 | 70 (19.9) |
|  | WHO 3-4 | 3 (0.9) |
|  | WHO 4 | 233 (66.2) |
| Marriage (%) |  | 1 (0.3) |
|  | Married | 340 (96.6) |
|  | Unmarried | 11 (3.1) |
